# Toxicity of Tioxazafen to *Meloidogyne Incognita* and *Rotylenchulus Reniformis*

**DOI:** 10.2478/jofnem-2022-0007

**Published:** 2022-04-17

**Authors:** Travis R. Faske, Katherine Brown, Jesse Kelly

**Affiliations:** 1 Division of Agriculture, Cooperative Extension Service, Lonoke Extension Center, University of Arkansas System, Lonoke, AR 72086

**Keywords:** behavior, EC_50_, hatch, nematicides, recovery, reniform nematode, southern root-knot nematode, toxicity

## Abstract

Tioxazafen is a seed-applied nematicide used in row crops. Currently, there are no data on nematode toxicity, nematode recovery, or effects of low concentrations of tioxazafen on nematode infection of a host root for *Meloidogyne incognita* or *Rotylenchulus reniformis*. Nematode toxicity and recovery experiments were conducted in water solutions of tioxazafen, while root infection assays were conducted on tomato. Nematode paralysis was observed after 24 hr of exposure at 27.0 µg/ml tioxazafen for both the nematode species. Based on an assay of nematode motility, 24-hr EC_50_ values of 57.69 µg/ml and 59.64 µg/ml tioxazafen were calculated for *M. incognita* and *R. reniformis*, respectively. Tioxazafen rates of 2.7 µg/ml and 27.0 µg/ml reduced the nematode hatch after 3 d of exposure for both the nematode species. There was no recovery in nematode motility after the 24-hr exposure of *M. incognita* and *R. reniformis* to their corresponding 48-hr EC_50_ values of 47.15 µg/ml and 47.25 µg/ml tioxazafen, respectively. Mortality of *M. incognita* continued to increase after 24 hr exposure, whereas *R. reniformis* mortality remain unchanged after nematodes were rinsed and removed for 48 hr from the tioxazafen solution. A 24-hr exposure to low concentrations of 0.38 to 47.15 µg/ml for *M. incognita* and 47.25 µg/ml for *R. reniformis* reduced the infectivity of each nematode species on tomato roots. The toxicity of tioxazafen was similar between nematode species; however, a greater rate of tioxazafen was needed to suppress *R. reniformis* infection of tomato than for *M. incognita*.

The southern root-knot nematode, *Meloidogyne incognita*, and the reniform nematode, *Rotylenchulus reniformis*, are two of the most important plant-parasitic nematodes that affect row crops in the United States (Sikora *et al*., 2018). In the southern US, *M. incognita* ranked first in lint yield loss in cotton (*Gossypium hirsutum*) and ranked second in grain yield losses in soybean (*Glycine max*) in 2019 (Lawrence *et al*., 2020; Bradley *et al*., 2021), while *R. reniformis* ranked second in cotton yield loss and ranked lower than 10 other diseases in soybean (Lawrence *et al*., 2020; Bradley *et al*., 2021). To mitigate yield losses due to these species, an integrated approach that consists of crop rotation, host plant resistance, and nematicides is often used.

Nonfumigant nematicides are among the most common nematicides used in row crop agriculture, with seed-applied nematicides being among the most popular. The application method is easy to use, reduces risk to human safety, and often uses less pesticide per hectare than nematicides applied in-furrow. Over the past 15 yr, there has been a general trend to market nonfumigant nematicides that have a lower risk to human safety and impact on non-target organisms. One nematicide marked with a lower risk to human safety and being evaluated for its effect on plant-parasitic nematodes is tioxazafen.

Tioxazafen is in the phenyl oxadiazole chemistry class and was registered in 2017 as a seed-applied nematicide (NemaStrike ST) by Bayer CropScience (formerly Monsanto Company, Chesterfield, MO) for use in corn (*Zea mays*), soybean, and cotton. The mode of action of tioxazafen involves a disruption of ribosomal activity in nematodes (Jewett and Koper, 2016). A few studies have evaluated the field efficacy of tioxazafen with a wide variation in the suppression of *M. incognita* infection and yield protection (Faske *et al*., 2019, 2020; Ernest *et al*., 2020; Groover *et al*., 2020). Tioxazafen is nematicidal against plant-parasitic and free-living nematodes (Slomczynska *et al*., 2015); however, no data are available on the sensitivity or behavioral effects of *M. incognita* or *R. reniformis* to tioxazafen. Given the recent registration of tioxazafen for nematicidal use and lack of nematode response data availability, additional data on the sensitivity of target species is needed.

The objectives of this study were to: (i) characterize the toxicity of tioxazafen to *M. incognita* and *R. reniformis*, (ii) determine if the effects of tioxazafen on each nematode species are reversible, and (iii) determine the effect of low concentrations of tioxazafen on the infectivity of each nematode species.

## Materials and methods

### Nematode inoculum

*M. incognita* and *R. reniformis* were isolated originally from cotton and maintained on tomato (*Solanum lycopersicum* ‘Rutgers’) and cotton (‘DP 1646 B2XF’), respectively. Eggs of *M. incognita* and *R. reniformis* were collected from roots with 0.5% NaOCl and only freshly collected eggs were used (Hussey and Barker, 1973). Second-stage juveniles (J2) of *M. incognita* were collected in a hatching chamber (Vrain, 1977) and 24-hr-old J2 were used. Mixed life-stages of *R. reniformis* were extracted from infested soil using a modified Baermann funnel method and were collected with a 25-μm-pore sieve after 48 hr and used immediately.

### Effect of tioxazafen on nematode motility

*M. incognita* and *R. reniformis* were treated for 2 hr, 24 hr, and 48 hr with water solutions of 27.03 µg/ml, 2.70 µg/ml, 0.27 µg/ml, 0.03 µg/ml, and 0.0 µg/ml of tioxazafen. Agricultural grade tioxazafen (NemaStrike ST 4.51 SC, Bayer CropScience, Research Triangle Park, NC) was used in this study. These experiments were performed in 24-well Falcon tissue culture plates (Corning Life Science, Tewksbury, MA); each well received 500 µl of a ×2 concentration of the test solution, which contained 30 to 40 nematodes in 500 µl of distilled water. Incubation temperatures of 28°C for *M. incognita* and *R. reniformis* were maintained throughout these experiments. Motile and immotile nematodes were determined visually during each sample time with an inverted compound microscope (Axio Vert.A1, Carl Zeiss Microscopy, Thornwood, NY). Each treatment was replicated four times in three experiments for each nematode species, and the percentage of immotile nematodes was calculated for each species to determine the effective concentration response.

### Effect of tioxazafen on nematode hatch rate

Freshly collected *M. incognita* and *R. reniformis* eggs were treated for 3 d with water solutions of tioxazafen at the same rates and with the same materials used in the motility assay. The number of J2 that hatched from eggs was recorded daily and the percent hatch per day was determined. Each treatment was replicated four times, and the experiment was conducted twice for each nematode species.

### Reversible effect of tioxazafen

A large population (i.e., 2,000) of *M. incognita* or *R. reniformis* were treated for 24 hr with water solutions respective to their 48-hr EC_50_ values of tioxazafen, poured over a 25-μm-pore sieve, and rinsed twice with distilled water. Rinsed nematodes were transferred to a 24-well tissue culture plate containing distilled water. Nematodes treated with distilled water served as the negative control and nematodes treated for 48 hr to their corresponding 48-hr EC_50_ values of tioxazafen served as the positive control. Motile vs immotile nematodes were determined visually at 24 hr and 48 hr after rinse using an inverted compound microscope. Each treatment was replicated four times in the three experiments and the percentage of immotile nematodes was determined for each species.

### Effect of low concentrations of tioxazafen on nematode infectivity

The impact of tioxazafen on the infectivity of *M. incognita* and *R. reniformis* on tomato roots was evaluated in a greenhouse experiment. A large population (i.e., 4,000) of each nematode species was treated for 24 hr with water solutions of tioxazafen corresponding to their 48-hr EC_50_ (1:5 dilutions). Nematodes were inoculated onto the 2-wk-old tomato seedlings growing in sand in an 84-cm^3^ celled seedling tray. Each seedling received 2 ml of the tioxazafen solution containing 500 nematodes. The inoculum was distributed through three holes around the seedlings created by pushing a 1-ml pipette tip 3 cm into the root zone. Nematodes treated with distilled water served as the positive control. The experimental design was a randomized complete block design, each treatment was replicated six times, and the experiment was conducted twice per nematode species. Tomato plants were incubated at 28°C to 30°C on a greenhouse bench and sampled 3 wk after inoculation to determine infectivity. Galls per root system was used to determine infectivity by *M. incognita*. Females of *R. reniformis* were stained with acid fuchsin (Byrd *et al*., 1983) to aid in counting the total females per root system.

### Statistical analysis

Data from the repeated experiments were similar (*P* > 0.05) and combined for final analysis. For these preliminary analyses, experiment repetitions were modeled as a random variable. Data from the nematode motility experiments were subjected to probit analysis using SPSS 27.0 (International Business Machines Corp., Armonk, NY) to determine the effective concentration response (i.e., EC_50_ and EC_90_). Data from hatch rate experiments were analyzed using a factorial ANOVA in the general linear mixed (GLM) model procedure with treatment replications modeled as a random variable, and nematicide rates and sample time were modeled as fixed factors using SPSS 27.0. Data from infectivity and recovery experiments were subject to ANOVA in the general linear model procedure. Data from hatch, recovery, and infectivity assays were log (x + 1) transformed when appropriate to normalize for analysis, and non-transformed data are reported. Means were separated according to Fisher’s *least significant difference* (LSD) procedure (α = 0.05).

## Results

**Figure 1:**
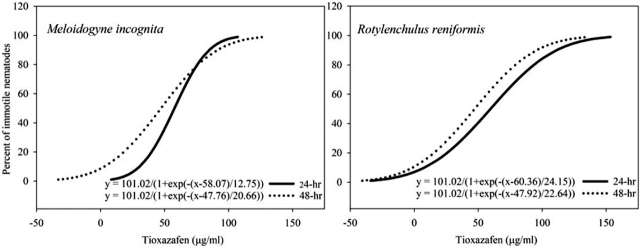
Relationship between the paralyses of *M. incognita* and *R. reniformis* treated for 24 hr and 48 hr with water solutions of tioxazafen. Equations were derived by nonlinear regression of probit analysis. For each equation, the *R*^2^ value was 0.99 (*P* = 0.0001).

*M. incognita* and *R. reniformis* were similar in sensitivity to tioxazafen. The onset of nematode paralysis was observed after 24 hr of continuous exposure for both nematode species. Based on the nematode motility assays, 8% of *M. incognita* were immotile after 24 hr at 27.0 µg/ml tioxazafen, while 22% of *R. reniformis* were immotile. Nematode paralysis increased for both species after 48 hr at 27.0 µg/ml tioxazafen to 37% for *M. incognita* and 31% for *R. reniformis*. The 24-hr EC_50_ values for tioxazafen were 57.69 µg/ml and 59.64 µg/ml for *M. incognita* and *R. reniformis*, respectively (Fig. 1). The increased duration of exposure did not have a drastic decrease in the concentration of tioxazafen needed to achieve a 48-hr EC_50_. The 48-hr value was 47.15 µg/ml for *M. incognita* and 47.25 µg/ml for *R. reniformis*. The EC_90_ values at 24 hr exposure for *M. incognita* and *R. reniformis* were 84.95 µg/ml and 111.30 µg/ml, respectively (Fig. 1).

**Figure 2:**
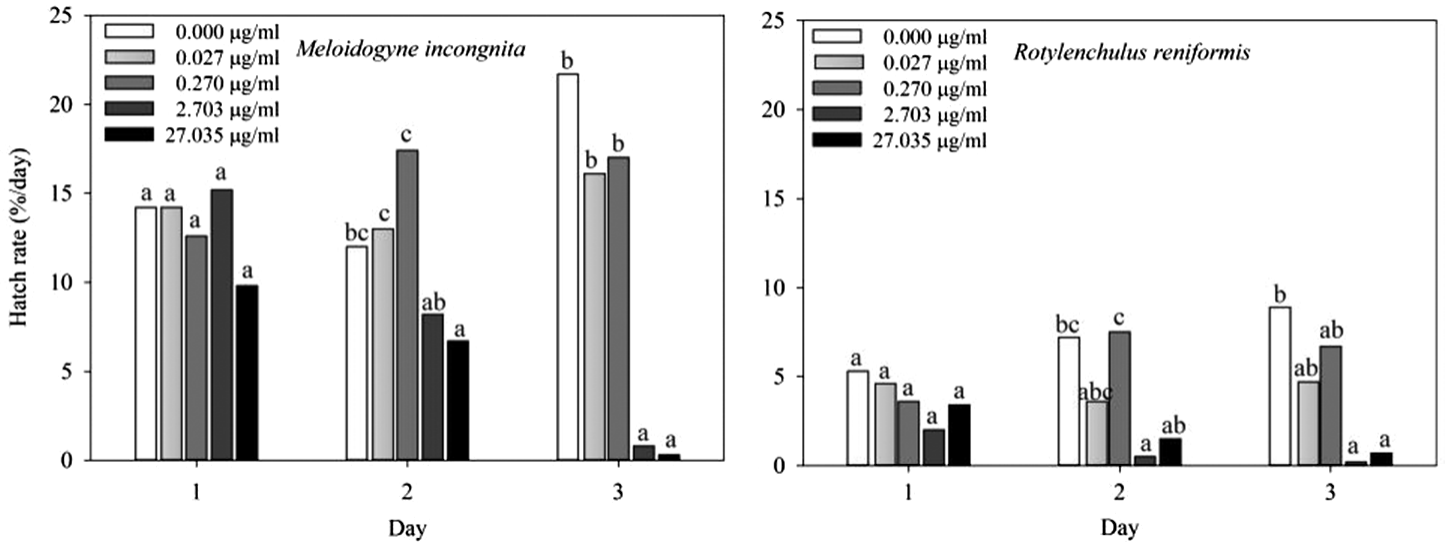
Effect of tioxazafen on hatch of *M. incognita* and *R. reniformis*. Different letters over bars per sample day indicate a significant difference at α = 0.05 according to Fisher’s LSD procedure. LSD, *least significant difference*.

Tioxazafen was somewhat effective in suppressing *M. incognita* and *R. reniformis* juvenile hatch rate (Fig. 2). There was a tioxazafen concentration by sample date interaction (*P* < 0.05) for both nematode species. A greater (*P* = 0.05) hatch suppression was observed at 2.7 µg/ml and 27.0 µg/ml after 3 d of continuous exposure compared to the water control for both the nematode species. Hatch was not affected by tioxazafen at concentrations <2.7 µg/ml for either nematode species.

**Figure 3:**
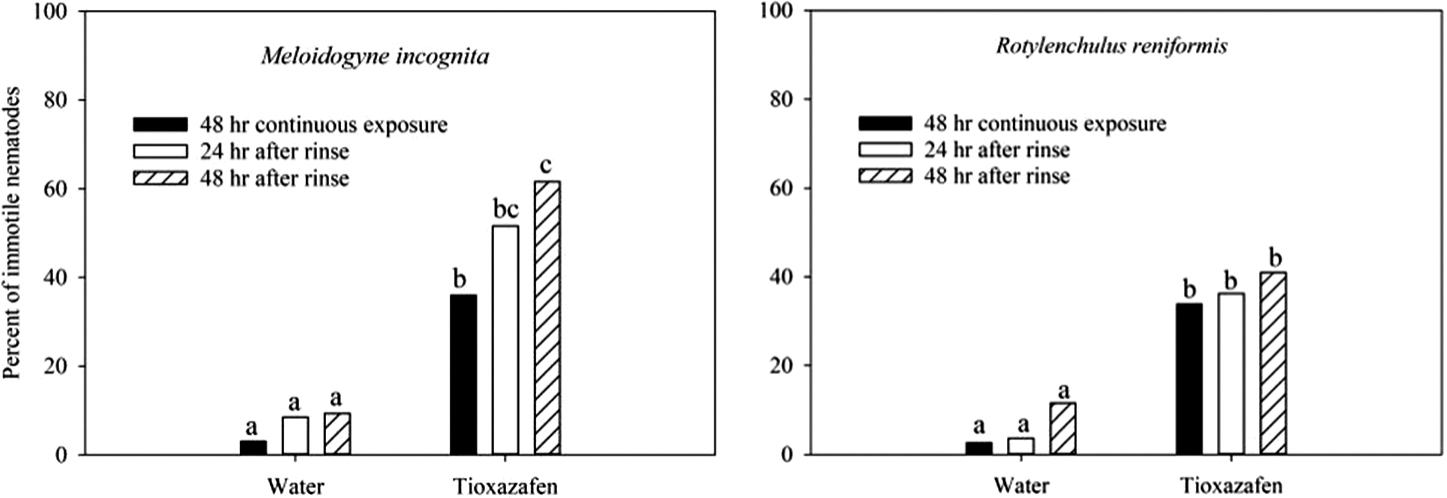
Recovery of *M. incognita* and *R. reniformis* treated with tioxazafen. Each species was treated with water solutions corresponding to its 48-hr EC_50_ value of tioxazafen for 24 hr, and then rinsed and transferred to distilled water. Different letters over bars indicate a significant difference at α = 0.05 according to Fisher’s LSD procedure. LSD, *least significant difference*.

No recovery in nematode motility was observed for *M. incognita* and *R. reniformis* when removed from tioxazafen after a 24 hr treatment to their corresponding 48-hr EC_50_ concentration (Fig. 3). There was an increase (*P* = 0.05) in the percent of *M. incognita* that were immotile 48 hr after being removed from the tioxazafen compared to the positive control, 48 hr of continuous exposure. A similar trend was observed with *R. reniformis*. Nematode posture for both species remained rigid when removed from the tioxazafen.

**Figure 4:**
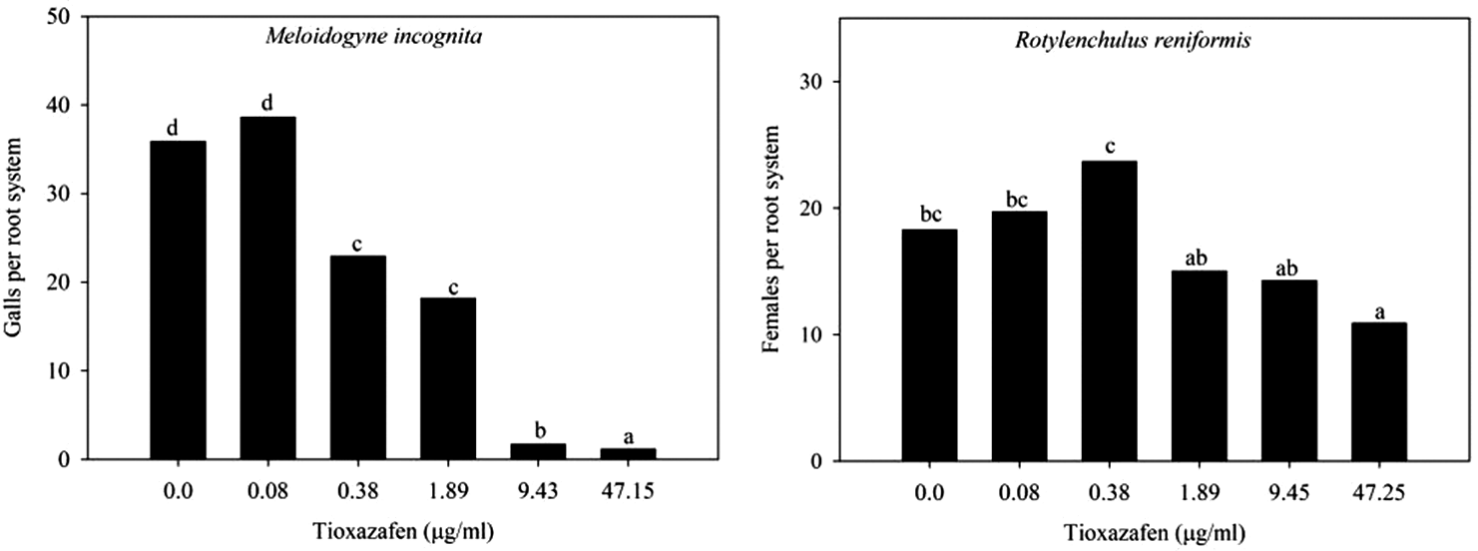
Effect of low concentrations of tioxazafen on infectivity of *M. incognita* and *R. reniformis* on tomato roots. Different letters over bars indicate a significant difference at α = 0.05 according to Fisher’s LSD procedure. LSD, *least significant difference*.

Low concentrations of tioxazafen were effective at reducing *M. incognita* but less effective at reducing *R. reniformis* infection of tomato roots. Rates of tioxazafen from 0.38 µg/ml to 47.15 µg/ml reduced (*P* = 0.05) root galling by *M. incognita* compared to the water control (Fig. 4). Only 47.25 µg/ml tioxazafen reduced (*P* = 0.05) the number of *R. reniformis* females observed per root system compared to the water control (Fig. 4).

## Discussion

Tioxazafen had a negative effect on the motility of *M. incognita* and *R. reniformis*, which was affected by the concentration and duration of exposure to the nematicide. The 24-hr EC_50_ value was 47.15 µg/ml tioxazafen for *M. incognita*, which is less toxic than other chemical nematicides used in cotton and soybean. For example, the 24-hr EC_50_ value for *M. incognita* was 1.2 µg/ml fluopyram and 0.42 µg/ml abamectin (Faske and Starr, 2006; Faske and Hurd, 2015). Similarly, the 24-hr EC_50_ value was 47.25 µg/ml for *R. reniformis*, which is less toxic than that reported for fluopyram, which is 1.97 µg/ml and for abamectin, which is 3.49 µg/ml (Faske and Starr, 2006; Faske and Hurd, 2015). These findings indicate that the toxicity of tioxazafen to *M. incognita* and *R. reniformis* is lower than that of fluopyram and abamectin, which are other seed-applied nematicides used for cotton and soybean.

Second-stage juvenile hatch for *M. incognita* and *R. reniformis* was suppressed after 3 d of exposure at concentrations of 2.7 µg/ml and 27.0 µg/ml tioxazafen. Fluopyram was reported to suppress the hatch of *M. incognita* but not *Caenorhabditis elegans* at 25 µg/ml (Heiken, 2017). Abamectin was reported to completely suppress the hatch of *Meloidogyne arenaria* at 10 µg/ml (Cayrol *et al*., 1993). These data support the nematicidal activity of tioxazafen to suppress hatch of *M. incognita* (Slomczynska *et al*., 2015) and expands to include *R. reniformis*.

Nematode paralysis was irreversible 48 hr after removal from the tioxazafen solution for both *M. incognita* and *R. reniformis*. Abamectin was reported to have irreversible effects on nematode paralysis, whereas fluopyram effects were reversible (Nordmeyer and Dickson, 1985; Faske and Hurd, 2015). The labeled rate of tioxazafen as a seed treatment ranges from 0.25 µg/seed to 1.0 µg/seed. Thus, the concentration on the seed coat is sufficient to impact nematode motility; however, field efficacy studies have reported limited suppression of *M. incognita* infection and yield protection in cotton and soybean (Faske *et al*., 2019, 2020; Ernest *et al*., 2020; Groover *et al*., 2020). Tioxazafen is slightly mobile in soil, which may impact soil movement, limit suppression of nematode infection, and subsequent protection of yield potential (Jewett and Koper, 2016).

Low concentrations of tioxazafen were more effective at inhibiting infection of tomato roots by *M. incognita* than that by *R. reniformis*. Sublethal concentrations of abamectin and fluopyram inhibited tomato root infection by *M. incognita* and *R. reniformis* (Faske and Starr, 2006; Faske and Hurd, 2015). There were differences in the sublethal effects of tioxazafen on *M. incognita* and *R. reniformis* on tomato, and this indicates that suppression of nematode infection may differ among plant-parasitic nematodes in response to tioxazafen.

The toxicity of tioxazafen is less than those of abamectin and fluopyram in vitro, and like abamectin, its effects on *M. incognita* and *R. reniformis* are irreversible. Since the 2020 cropping season, tioxazafen was voluntarily removed from commercial use by the manufacturer. Thus, these data serve as a reference for the toxicity of tioxazafen to two important yield-limiting plant-parasitic nematodes if it returns as a commercially available nematicide.
